# Small Heat Shock Protein αB-Crystallin Controls Shape and Adhesion of Glioma and Myoblast Cells in the Absence of Stress

**DOI:** 10.1371/journal.pone.0168136

**Published:** 2016-12-15

**Authors:** Miho Shimizu, Mikihito Tanaka, Yoriko Atomi

**Affiliations:** 1 Material Health Science Laboratory, Graduate School of Engineering, Tokyo University of Agriculture and Technology, Tokyo, Japan; 2 Graduate School of Arts and Sciences, The University of Tokyo, Tokyo, Japan; Institut de Genetique et Developpement de Rennes, FRANCE

## Abstract

Cell shape and adhesion and their proper controls are fundamental for all biological systems. Mesenchymal cells migrate at an average rate of 6 to 60 μm/hr, depending on the extracellular matrix environment and cell signaling. Myotubes, fully differentiated muscle cells, are specialized for power-generation and therefore lose motility. Cell spreading and stabilities of focal adhesion are regulated by the critical protein vinculin from immature myoblast to mature costamere of differentiated myotubes where myofibril Z-band linked to sarcolemma. The Z-band is constituted from microtubules, intermediate filaments, cell adhesion molecules and other adapter proteins that communicate with the outer environment. Mesenchymal cells, including myoblast cells, convert actomyosin contraction forces to tension through mechano-responsive adhesion assembly complexes as Z-band equivalents. There is growing evidence that microtubule dynamics are involved in the generation of contractile forces; however, the roles of microtubules in cell adhesion dynamics are not well determined. Here, we show for the first time that αB-crystallin, a molecular chaperon for tubulin/microtubules, is involved in cell shape determination. Moreover, knockdown of this molecule caused myoblasts and glioma cells to lose their ability for adhesion as they tended to behave like migratory cells. Surprisingly, αB-crystallin knockdown in both C6 glial cells and L6 myoblast permitted cells to migrate more rapidly (2.7 times faster for C6 and 1.3 times faster for L6 cells) than dermal fibroblast. On the other hand, overexpression of αB-crystallin in cells led to an immortal phenotype because of persistent adhesion. Position of matured focal adhesion as visualized by vinculin immuno-staining, stress fiber direction, length, and density were clearly αB-crystallin dependent. These results indicate that the small HSP αB-crystallin has important roles for cell adhesion, and thus microtubule dynamics are necessary for persistent adhesion.

## Introduction

Although αB-crystallin is categorized as a small heat shock protein (HSP) [1], growing evidence shows that αB-crystallin is a protein that is expressed ubiquitously under unstressed conditions. Both the αB-crystallin transgene [2] and αB-crystallin administration [3] were found to protect against cardiac injury. Other potential therapeutic applications of αB-crystallin include neuronal inflammation [4–7]. These protective roles may be related to proteostasis [8] because αB-crystallin exerts its functions under inflammatory conditions where denatured proteins may exist inside of cells.

αB-crystallin decreases in atrophied muscle during rat hindlimb suspension experiments [9] [10] that mimic bedridden patients or a microgravitational environment. Immunostaining shows that αB-crystallin colocalizes with several cytoskeletal [11] and focal adhesion proteins in muscle [12]. In muscle cells, αB-crystallin is preferentially expressed in slow-twitch muscle compared to fast-twitch muscle [9, 13] and this may be correlated with higher mitochondrial numbers and elevated oxidative stress and protein turnover rate in type I fibers [14]. Muscle fiber types are generally distinguished by the predominant myosin heavy chain isoforms present in the particular muscle. Dysfunction of mitochondria is a typical phenomenon during muscle aging accompanied by accumulations of ROS and lipid/protein damage [15] where chaperon function and sequestrations of denatured proteins by autophagy/ubiquitin–proteasome system is necessary but attenuated.

αB-crystallin localizes to the wide z-band of the sarcomere where mechanical contractile tension is exerted by the actomyosin system [10, 16], and it may protect cytoskeletal proteins from mechanical stress [12, 16]. Muscle atrophy and hypertrophy have been studied for many years using myoblast cells as a model system [17, 18]. Previously we have shown that αB-crystallin also has a role in myoblast differentiation and αB-crystallin-deficient C2C12 myoblast cells failed to form myotubes [19]. The fly ortholog of αB-crystallin is required for muscle structural integrity and function [20].

Oxidative stress occurs in muscle cells as well as glial cells in the brain. Chronic oxidative stress in the brain leads to the accumulation of aggregated protein products that are characteristic of neurodegenerative pathology such as Alzheimer's disease. αB-crystallin is constitutively expressed in glial cells (S1 Fig) where it contributes to brain homeostasis over a lifetime [21]. Recent findings revealed that glial cells fuel neurons by glycolysis [22], sequester ROS-induced peroxidized lipids in the brain for neuroblast protection [23], generate respiratory rhythms both in normoxic and hypoxic conditions [24] and clear metabolites during sleep [25]. Since both muscle and glial cells constitutively express αB-crystallin where oxidative metabolism is high, there is likely a common cellular function. Here, we attempted to establish the nature of that function.

In this study, we used glial and myoblast cell lines in which αB-crystallin was overexpressed or knocked down. We found that αB-crystallin knockdown cells were highly motile as revealed by time-lapse observation. This may be due to limited cell adhesion because of fragile microtubule dynamics without αB-crystallin chaperon activity. On the other hand, overexpression of αB-crystallin led to a fully extended cytoskeletal structure with a relative immotile phenotype. During muscle contraction, it is well known that cells are exposed to oxidative stress, but no study has focused on cell shape and migration as related to tubulin/microtubules in the cytoskeleton. Heart muscle contracts at a slow frequency, which is thus more stressful compared to skeletal muscle and αB-crystallin seems to play an important protective role not only for FAK degradation by calpain [12] but also for tubulin cytoskeleton (Ohoto-Fujita and Atomi, manuscript in preparation). In this study we focused on the physiological function of αB-crystallin, as a ubiquitously expressed protein [26], in the absence of stress. We hypothesize that it has a fundamental role in the maintenance of cell shape and adhesion, roles that have been overlooked until now.

## Materials and Methods

### Cell lines

The C6 rat glioma cell line was a generous gift from Dr. T. Iwaki, Kyushu University, Japan [27]. The L6 rat skeletal myoblast cell line was obtained from the American Type Culture Collection. C6 cells were cultured in F10 (Gibco BRL, Rockville, MD), and the other cells were cultured in DMEM (Gibco BRL, Rockville, MD) both supplemented with 10% fetal bovine serum (FBS) (GIBCO-BRL, Life Technologies, Inc., Rockville, MD, USA) and 80 μg/mL kanamycin (Meiji Seika, Tokyo, Japan). All cell lines were maintained at subconfluent densities (60–70%) and were incubated at 37°C in 5% CO_2_ in a humidified chamber.

### Preparation of cells overexpressing αB-crystallin or in which αB-crystallin was knocked down

Rat αB-crystalline-plasmids were a generous gift from Dr. T. Iwaki, Kyushu University, Japan [27]. αB-crystallin-overexpressing cell lines were prepared by co-transfection of pSV-neo (neomycin resistant) and pRSV-SSE (which includes the αB-crystalline-sense coding region). αB-crystalline knockdown cell lines were obtained by the co-transfection of pSV-neo and pRSV-SAS (which includes an αB-crystallin-antisense coding region). Plasmids (pSV-neo: pRSV-SSE or AS = 1: 10) were introduced into the cells by electroporation using a CUY-21 electroporator (TR-Tech, Tokyo, Japan). For the control (wild-type) cell lines, only the pSV-neo plasmid was introduced. Successfully transfected cells were selected by their neomycin resistance by adding Geneticin to the culture media to a final concentration of 400 μg/mL (G-418, Life Technologies, Inc., Rockville, MD, USA). Single clones were picked up after 2 weeks and αB-crystallin expression levels were assessed by Western blotting (S2 Fig). Individual clones were maintained in medium containing 200 μg/mL Geneticin to prevent plasmid loss. Established cell lines used for all of the experiments included control (wild-type), αB-crystalline overexpressing (OE) cells and αB-crystalline knockdown (KD) cells.

### Time-lapse studies

Viable cells were plated on glass-bottomed dishes (Iwaki, Tokyo Japan) and were observed under an inverted fluorescent microscope (Axiovert 135TV, Carl-Zeiss, Oberkochen, Germany) equipped with Plan-Neofluor 10x, 20x and 40x objectives. Time-lapse micrographs were acquired using a Sensys CCD digital camera (Photometrics, Tucson, AZ, USA) attached to the microscope. For extended image capturing, we used a handmade incubation system that maintains cells under appropriate conditions for culture (37°C, 5% CO_2_). HEPES 10 mM (pH 7.2) (final) was added to the culture media to maintain neutral pH during observation. Each dish was photographed up to 2 h at 3 min intervals. The time-lapse images were analyzed using IPLab Spectrum software (Scanalytics, Fairfax, VA, USA.). Cell migration speed was calculated as the distance that the center of gravity of the cell contour moved in each time interval. The shape index (SI) was calculated from the cell contour and indicates the deformation of the shape. The SI of a circle is 1. The formula to obtain SI is as following: [SI] = 4πA/P^2^ (A: area, P: Perimeter) [28].

### Cell proliferation and cell cycle analysis

C6 wild-type, αB-crystalline overexpressing and αB-crystalline knockdown cells were grown on 10 plates. A set of plates was removed from the incubator for either time-lapse observation or ethanol fixation and propidium iodide staining for flow cytometric analysis (EPICS XL, Coulter) at five different time points when the cell density reached 10%, 25%, 50%, 80%, and 95%.

### Drug treatment

The following cytoskeletal targeting drugs were used: Nocodazole (10 μg/mL final concentration, Sigma) for tubulin depolymerization, Paclitaxel (20 μM final conc., Sigma) for tubulin stabilization, and cytochalasin-D (CyD, 0.02 μM final conc., GIBCO BRL) for actin depolymerization. Cells were cultured on a microscope stage for 2 h before adding drugs to the medium.

### Microinjection

N-terminal (N1) or C-terminal (C1) anti-αB-crystallin antibody and purified non phosphorylated form of αB-crystalline protein from bovine lens were prepared as described previously [11] and those were injected into the cells with an Eppendorf injection system (Transjector #5246, Micro manipulator #5171). Cells were cultured on Cellocate (Eppendorf) grid-etched glass plates for 36 h before injection. N1/C1-terminal antibodies were diluted 100-times in PBS and injected into αB-crystallin-overexpressing C6 cells. Ten pg of purified αB-crystallin protein was injected into a single αB-crystallin knockdown C6 cell. Alexa-Fluor 488(Abs: 495nm, Em: 519 nm; Molecular Probes) in PBS was co-transfected into the cells as an injection marker. After injection, the culture dish was placed in an incubator on an Axiovert 135TV microscope stage, and both translucent and fluorescent time-lapse pictures were recorded.

### Transfection of Rac1 plasmids

Expression vectors, Rac1 (wild-type), Rac1^V12^ (constitutively active), and Rac1^N17^ (dominant negative) mutants conjugated with EGFP (Enhanced Green Fluorescent Protein) were a kind gift from Dr. K. Kaibuchi (Nagoya Univ.). Plasmids were introduced into C6 wild-type, αB-crystallin-overexpressing, and αB-crystallin knockdown cells using Effectene Transfection Reagent (Qiagen).

### Immunofluorescence study

Cells were fixed and stained as previously described [11]. Briefly, cells were twice washed with Microtubule stabilizing buffer (MSB; 100 mM PIPES-NaOH (pH 6.8), 1 mM MgCl_2_ and 1 mM EGTA) followed by MSBT (MSB+0.5% Triton-X 100) for 10 sec. at 37°C, then fixed in a 10% neutral buffered formalin (SIGMA) supplemented with 2 mM MgCl_2_, 2 mM EGTA, and 0.03% Triton-X 100 for 4 min at 37°C then room temperature for 6 min. Cells were washed with PBS for three times then store in a blocking solution (1% BSA, 0.02% NaN_3_ in PBS) until use at 4°C. Following antibodies and reagents were used for immunofluorescence; Monoclonal anti-Vinculin (V9131, SIGMA), Goat anti-Mouse IgG (H+L) Secondary Antibody, Alexa Fluor® 546 (A11030, Molecular Probes), Alexa Fluor® 488 Phalloidin (A12379, Molecular Probes), Monoclonal Anti-β-Tubulin−Cy3 antibody (clone TUB2.1, Sigma-Aldrich), and Hoechst 33342 (H3570, Molecular Probes) for DNA staining. Confocal microscope (Nikon A1RMP) was used for data aquisition. Adobe Photoshop CC (2015) was used for inverted contrast image.

### Statistical Analysis

Data are expressed as means ± standard error of the mean (s.e.m.) for a given number of observations. Comparisons between two normally distributed groups were made using an unpaired Student's *t*-test. Where appropriate, one way analysis of variance (ANOVA) was used to compare multiple groups. A P value of <0.05 was considered statistically significant.

## Results

### αB-crystallin protein-dependent alteration of cell morphology and other characteristics

**Fig 1:**
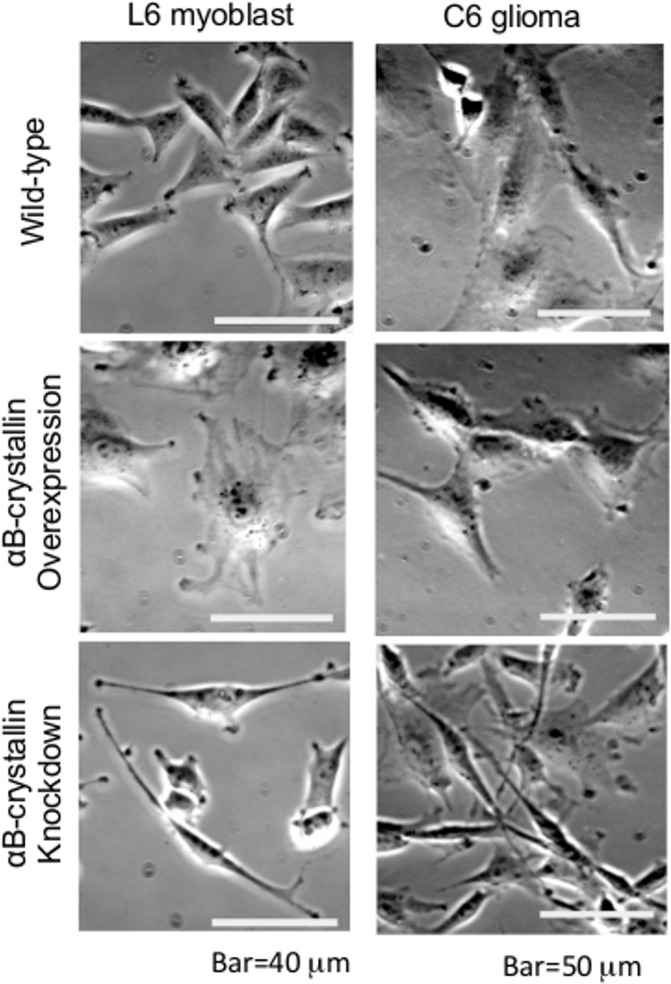
Phase-contrast images of αB-crystallin-overexpressing and knockdown L6 myoblasts, and C6 glioma cells. Compared with wild-type cells, αB-crystallin-overexpressing cells had a more spread shape and knockdown cells showed a narrow, fibroblast-like shape in both cell types.

There is growing evidence that αB-crystallin associates with cytoskeletal fibers in dynamic assembly studies using purified proteins [11, 29, 30] as well as in cell lines [11, 19, 31] and primary glial cells (S1 Fig). Therefore, we first focused on cell shape to see if there were αB-crystallin-dependent morphological alterations. Both αB-crystallin-overexpressing and knockdown cell lines were prepared and analyzed (S2 Fig). Compared with wild-type cells, αB-crystallin-overexpressing cells had a more rounded and spread shape (Fig 1). Since αA- and αB-crystallin has been well known for affecting cell proliferation kinetics in lens epithelial cells [32], cell doubling time was examined in our experiment because microtubule stability may have altered the timing of cell cycle phase therefore proliferation. Data indicated about two- to three-fold longer for αB-crystallin-overexpressing cells: 52.2 hr for C6 (24.3 hr for wild-type C6), and 84.2 hr for L6 (23.8 hr for wild-type L6). Both C6 and L6 αB-crystallin-overexpressing cells had wide filopodia and highly-developed ruffled membranes and therefore cells spread extensively, revealing the presence of stabilized microtubules (S3 Fig). αB-crystallin knockdown cells showed a characteristic shape. Typically, cells possessed a narrow, fibroblast-like shape with extensively lengthened pseudopodia (Fig 1 and S3 Fig). C6 αB-crystallin knockdown cells had a stronger phenotype compare to L6 cell lines (S3 Fig). The longest pseudopodia reached 300 μm (data not shown). This elongated but thicker, fibroblast-like phenotype (S3 Fig) was also confirmed by a dsRNAi method (S4 Fig). Doubling-time was relatively longer than wild-type for both C6 (49.2 hr) and L6 cells (37.4 hr). The characteristic altered cell morphology of overexpressing as well as knockdown cells was significantly different from wild-type (<0.005) when more than 20 cells were assessed for each population.

### Phenotype of cells overexpressing αB-crystallin or lacking αB-crystallin was morphologically reversible

**Fig 2:**
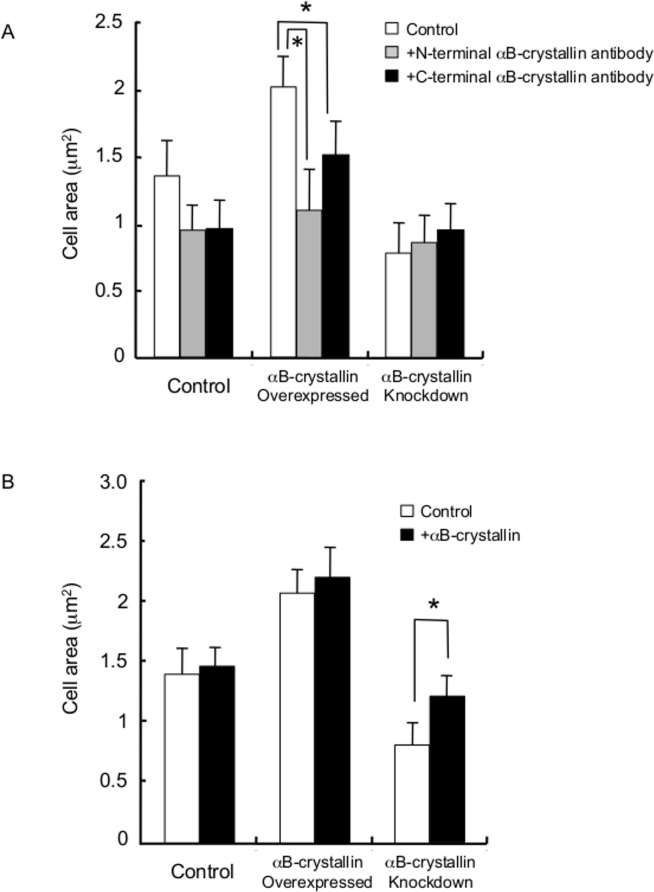
Cell morphology and αB-crystallin level. (A) Characteristic cell morphology is specific to αB-crystallin level as revealed by inhibitory antibody injection and (B) injection of purified bovine lens protein αB-crystallin into wild-type, αB-crystallin-overexpressing and knockdown C6 cells. Asterisk indicates P value of <0.05.

To confirm that the characteristic cell morphology was specific to αB-crystallin, anti-αB-crystallin antibody was introduced into αB-crystallin overexpressing C6 cells by a microinjection technique. Antibodies raised against both N- and C-terminal αB-crystallin protein were injected into overexpressing cells. We found that targeted inhibition of the specific protein showed a knockdown-like phenotype (S5 Fig). Antibody-injected overexpressing cells showed a significantly reduced cell area (P<0.05) (Fig 2A), and in particular, cells injected with the N-terminal antibody showed loss of the spreading phenotype and adopted a fibroblast-like morphology after five h (S5 Fig). On the other hand, C-terminal antibody-injected overexpressing cells possessed lengthened pseudopodia (S5 Fig). Purified bovine lens αB-crystallin protein-injected knockdown cells became phenotypically wild-type (S5 Fig); that is, we observed a significant cell area increment (Fig 2B) and recovery of the spreading phenotype (asymmetric ruffled-membrane production) (S5 Fig). In summary, the extended radial morphology produced by αB-crystallin overexpression was reversed to that of wild-type by αB-crystallin antibody injection and elongated fibroblast-like morphology due to knockdown was reverted to wild-type, although it involved a multi-step cytoskeletal rearrangement by αB-crystallin protein injection. Thus, the alteration of cell morphology in both overexpressing and knockdown cells was αB-crystallin-specific.

### Characteristics of cell populations and their proliferation

**Fig 3:**
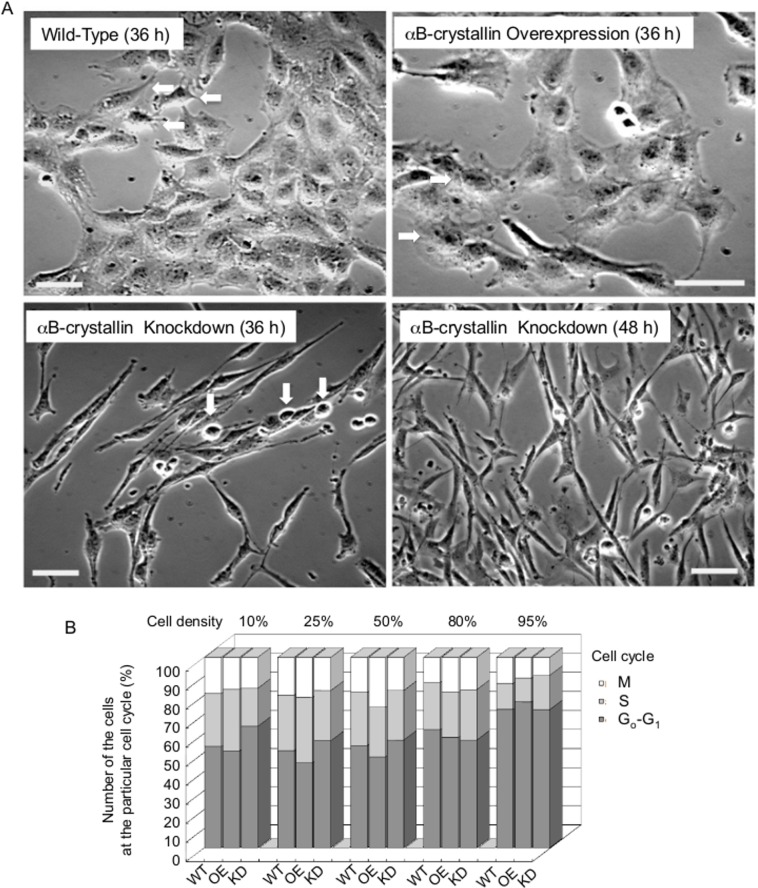
Morphology-dependent cell proliferation study of αB-crystallin-overexpressing cells and knockdown C6 cells. See detail in the text. Bar = 40 μm in A.

To gain insight into αB-crystallin-dependent cell morphology and function, the cells’ proliferative characteristics were examined at different cell densities. Phase-contrast live images were observed after 36 h or 48 h of growth for C6 wild-type, αB-crystallin-overexpressing cells and αB-crystallin knockdown cells after they were seeded at 10% confluence on culture dishes. In C6 wild-type, cells at the bottom right half of Fig 3A became confluent and contact inhibition was observed. Arrows point to actively moving cells where filopodia were significant and they are thick (seen as dark shadow). In αB-crystallin-overexpressing cells, lamellipodia and multinucleated cells (identified by arrows) were characteristic. Once the cells divided, they became fused into multinucleated cells (four nuclei maximum). In αB-crystallin knockdown cells at 36 h, characteristic spindle-shaped cells and significant filopodia were seen. Cells identified by arrows were not mitotic cells but partially detached from the substrate because of the cells’ overlap. After 48 h, knockdown cells had proliferated and stacked up on each other without contact inhibition and cells reached 95% confluence. Stacked knockdown cells were easily detached from the substrate and most of the trypsin-treated cells could not attach to the dish, floating in the culture medium where they eventually died. This response of knockdown cells is explained by the fact that they could only adhere to the substrate at the tips of the spindle-shape structures. When cells were treated with trypsin, the cell body detached easily but not the tips. Abnormal cytokinesis was also observed. Interestingly, active exocytosis was observed at the tips (data not shown). Cell cycle analysis of C6 cells was performed using flow cytometry. The tendency of the right shift of S-phase cells indicated a delay of DNA synthesis in knockdown cells (S6 Fig). The number of cells either at G_0_/G_1_-phase, S-phase, or M-phase was compared at five different cell density points (Fig 3B). Over a rather broad range of cell densities (25%, 50%, and 80%), αB-crystallin-overexpressing cells showed a higher proportion of M phase cells, which may explain the presence of multinucleated cells observed in Fig 3A. At 10%, 25% and 50% cell densities, the majority of the knockdown cell population was at the G_0_/G_1_-phase, consistent with a delayed M phase (S6 Fig).

### Cell shape changed by drug treatment

**Fig 4:**
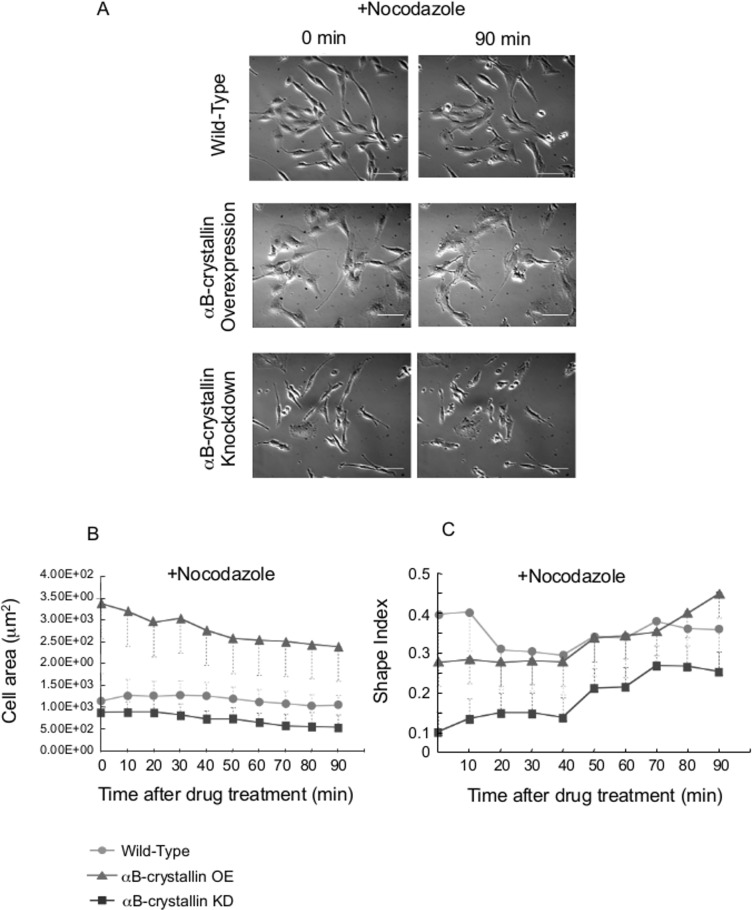
αB-crystallin-overexpressing cells and knockdown C6 cells: shape change after Nocodazole treatment. Images from just after the drug addition (final 10 μg/mL; 0 min, left panel) and after 90 min (right panel) (A). Bar = 40 μm. Cell area (B) and Shape index (C) change of C6 cells after Nocodazole treatment. N = 20.

Adaptation to the environment is an essential characteristic of the cell for survival. Among the many factors involved in the process, a balanced and coordinated actin-tubulin system is key for cellular homeostasis. Previously we found that αB-crystallin is a molecular chaperon for tubulin/microtubules through MAPs. We next assessed the level of αB-crystallin expression and its impact on cell shape by treating cells with drugs targeting cytoskeletal elements to alter their morphology. C6 glioma cells are differentiated malignant cells from the brain (neuro-epithelial origin) and are susceptible to various drug treatments. Nocodazole treatment inhibits polymerization of tubulin in the cell, and knockdown cells showed dramatic changes in both cell area and shape. This was consistent with the notion of its protective role (Fig 4A) (Fujita et al., 2004). Interestingly Nocodazole-treated αB-crystallin-overexpressing cells showed a reduced cell area compared to non-treated cells and they became compacted (Fig 4B and 4C). This can be attributed to an accumulation of Nocodazole-decorated tubulin/microtubules in the cell due to accelerated microtubule dynamics with a higher level of αB-crystallin compared to wild-type C6 glioma cells.

**Fig 5:**
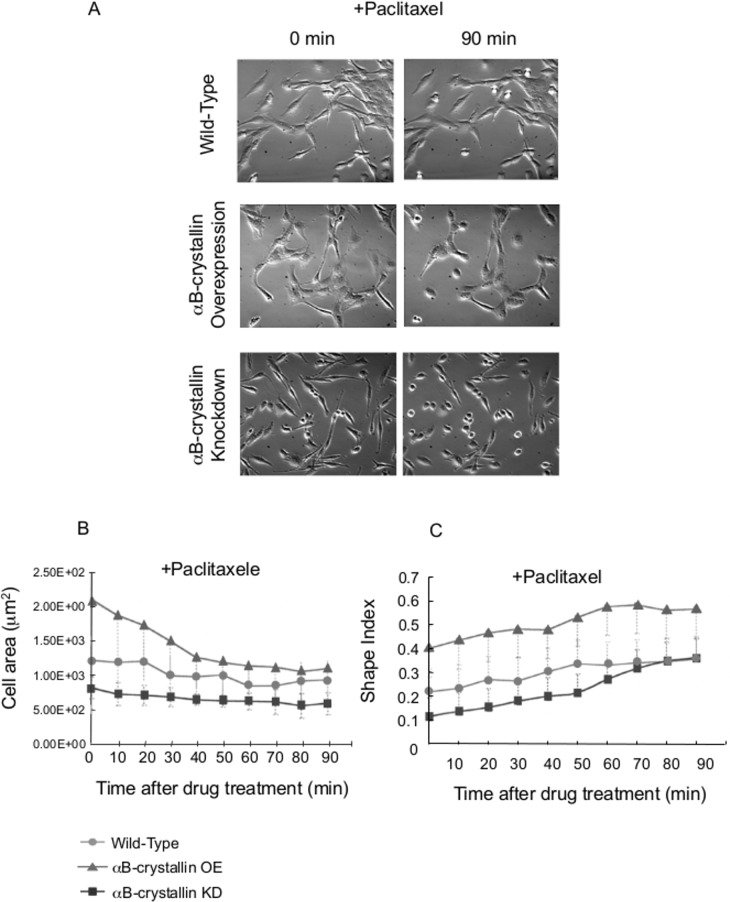
Effect of paclitaxel on αB-crystallin-overexpressing C6 cells and knockdown cells. Cell shape change after paclitaxel treatment. Just after the addition of drug (final 20 μM; 0 min, left panel) and 90 min later (right panel) (A). Cell area (B) and Shape index (C) change of C6 cells after paclitaxel treatment (final 20 μM). N = 20.

The susceptibility of glioma cells to anticancer drugs is important information for neoplastic treatment. Induced neural differentiation of paclitaxel-treated C6 glioma cells through cell morphologic changes with expression of neural markers was recently reported [33]. Extended morphology of αB-crystallin-overexpressing cells but not αB-crystallin knockdown cells was dramatically reduced by a microtubule stabilization agent, paclitaxel, indicating that stretched morphology is dependent on αB-crystallin-assisted microtubule dynamic instability (Fig 5). Elongated cell rods of knockdown cells were also found to be dynamic-microtubule-dependent (Fig 5C).

**Fig 6:**
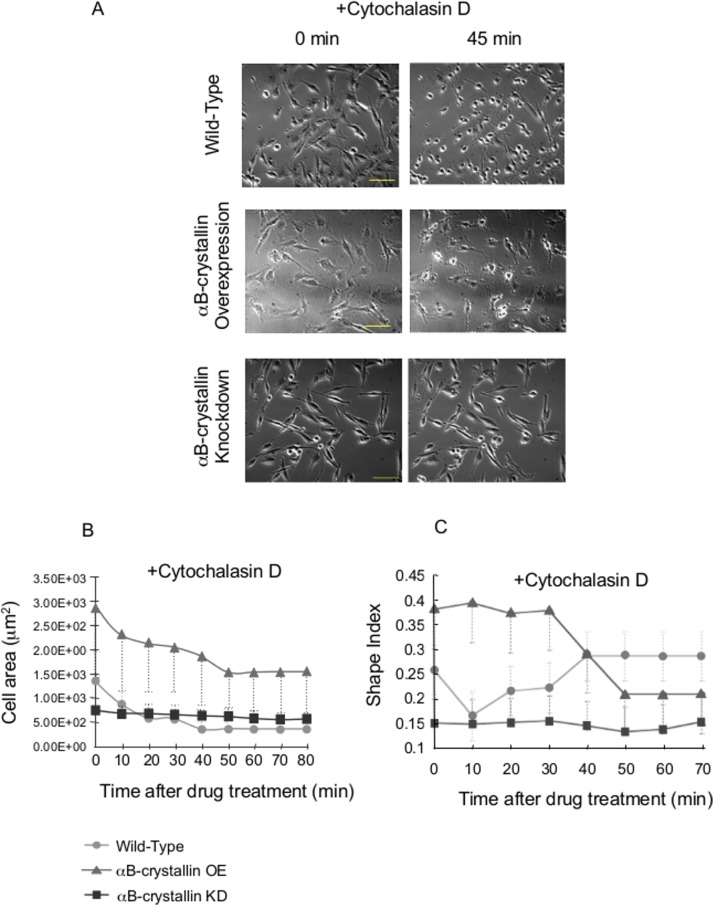
Effect of cytochalasin D on C6 αB-crystallin-overexpressing and knockdown cells. Just after drug addition (final 0.4 μM; 0 min, left panel) and 45 min (right panel) (A). Cell area (B) and Shape index (C) change of C6 cells after Cytochalasin D (final 0.4 μM). N = 20.

Cytochalasin D inhibits actin polymerization. Kobayshi reported that Cytochalasin D treatment of C6 glioma cells induced an arborized phenotype through formation of spider-like actin rearrangement [31]. As shown in Fig 6, both wild-type and αB-crystallin-overexpressing cells showed a similar spider-like phenotype (Fig 6A) and cell area reduction (Fig 6B). Morphological changes of overexpressing cells were more dramatic than wild-type, consistent with the involvement of αB-crystallin in actin polymerization as reported previously [27].

### Cell attachment area and morphology were Rac1-dependent in αB-crystallin-deficient cells

**Fig 7:**
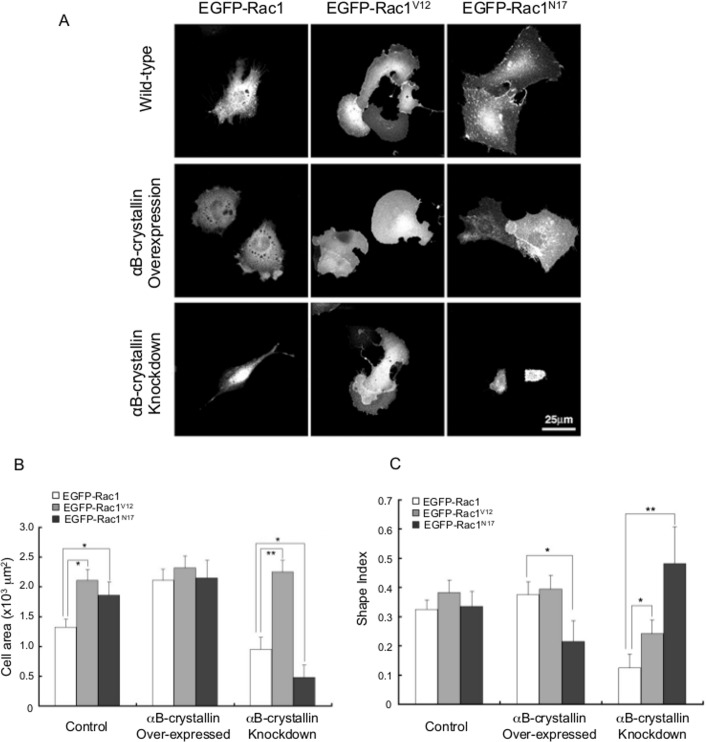
Impact of Rac1 on morphology of αB-crystallin-overexpressing and knockdown cells. C6 cells were transfected with EGFP-Rac1 (wild-type), EGFP-constitutively active Rac1 (V12), and EGFP-dominant-negative Rac1 (N17), and fluorescence images were taken after 12 h. αB-crystallin knockdown C6 cells that expressed Rac 1^V12^ showed enlarged peripheral ruffles at both ends of spindle-shaped cells. On the other hand, complete loss of lengthened pseudopods was observed in Rac1^N17^ transfected cells (A). Area change (B) and shape index (C) of EGFP-Rac1, Rac1^V12^, Rac1^N17^ expressed wild-type, αB-crystallin overexpressed, and knockdown C6 cells. ** = P<0.01, *P = 0.05, n = 50.

Since peripheral membrane ruffling caused by overexpression of αB-crystallin is typical for Rac1 activation, both constitutively active (V12) and dominant negative (N17) Rac1 co-expression were examined. All Rac1^V12^ -expressing cells showed ruffled membranes that were typical for Rac1 signaling (Fig 7). In the case of αB-crystallin knockdown cells, Rac1^V12^ expression doubled the cell attachment area, and this was reduced to one-half by Rac1^N17^. Although the exact mechanism by which αB-crystallin affects cell shape is not clear, both active and negative forms of Rac altered the αB-crystallin knockdown phenotype. Thus, there must be dynamic cytoskeletal protein scaffold structures that include focal adhesion complexes, actin, intermediate filaments, microtubules and their linking protein, plectin.

### αB-crystallin knockdown inhibited persistent cell adhesion

**Fig 8:**
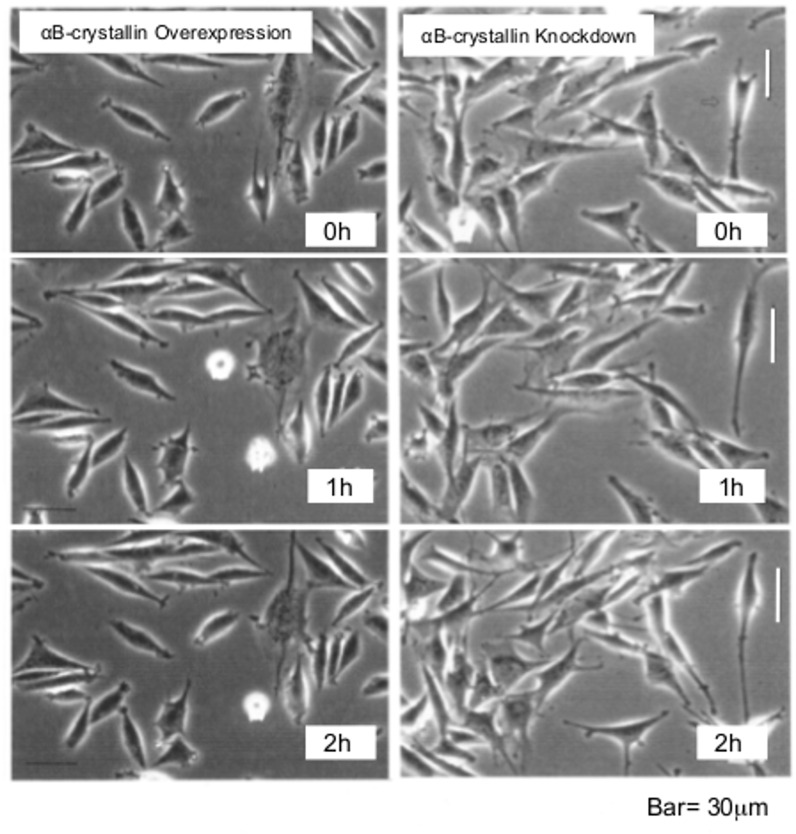
Time-lapse imaging. αB-crystallin-overexpressing cells (left column), and knockdown (right column) L6 cells at 0 h, 1 h, and 2 h time points. Bar is 30 μm.

**Fig 9:**
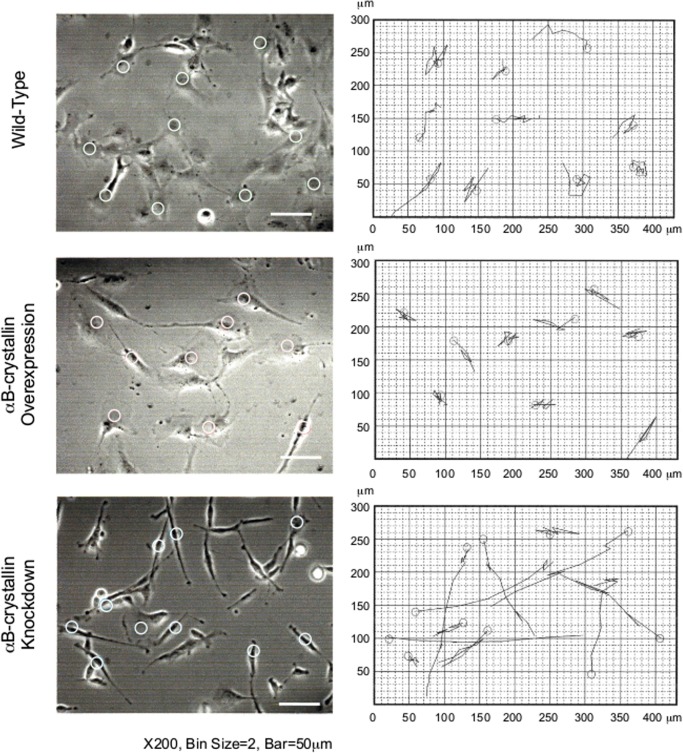
Mode of migration of wild-type, αB-crystallin overexpressing and knockdown cells. Bar is 50 μm. Time-lapse images were recorded for 2 h at 10 min intervals and traces of the migration were drawn on the right hand graph. Circles on the left panel indicate the center of the cells at the beginning of the image recordings.

**Fig 10:**
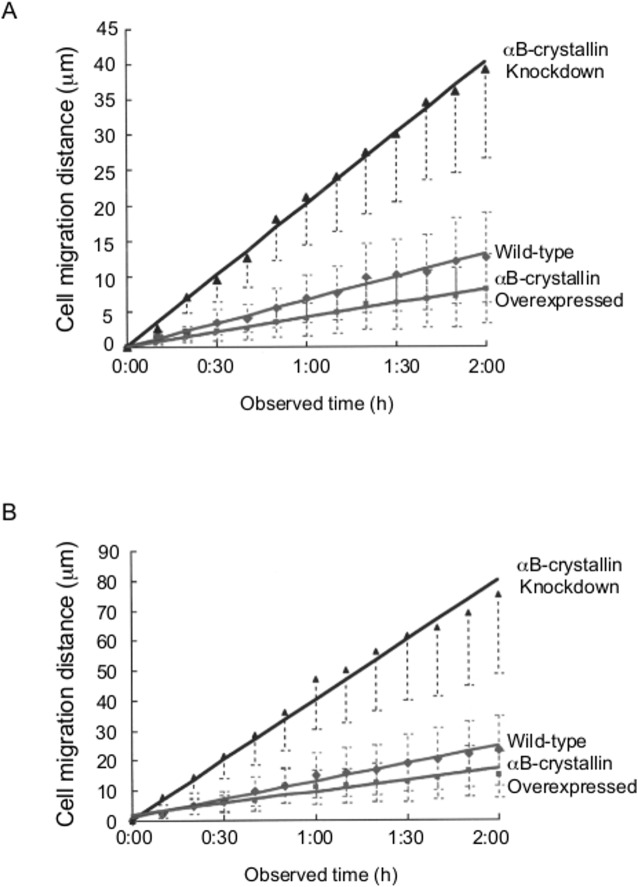
Time-dependent migration of wild-type, αB-crystallin-overexpressing and knockdown L6 myoblast cells (n = 200). Cumulative distances moved were obtained from images captured every 10 min (A). Time-dependent migration of wild-type, αB-crystallin-overexpressing and knockdown C6 glioma cells (n = 300). Cumulative moving distances were obtained from images captured every 10 min (B).

Time-lapse images of cells in which αB-crystallin was overexpressed (left column) or knocked down (right column) were recorded at 0 h, 1 h and 2 h time points (Fig 8). Typically, both small and extended cells were observed in αB-crystallin-overexpressing populations. Cells with active ruffled membranes and dynamic pseudopodia and pseudo M-phase rounded cells that never divided to restore their shape were characteristic of αB-crystallin-overexpressing cells. In knockdown populations, cells were less spread, with weak contact inhibition and extended pseudopodia. Time-lapse images were recorded for 2 h at 10 min intervals and traces of the migration were drawn on the right hand graph. For this study, we used 10 randomly chosen cells from each cell line (except for those cells that moved out of the frame within the time of observation) (Fig 9). Circles on the left panel indicate the center of the cells at the beginning of the image recordings. Note that the image on the left and graph on the right use different aspect ratios. Migration speed and distance were measured, and the correlation between them was plotted (Fig 10). For both myoblast cells (Fig 10A) and glioma cells (Fig 10B), knocked down cells migrated two to three times faster compared to wild-type cells. The specificity of this fast migration phenotype was examined. Migration speed reverted to wild-type levels when overexpressing C6 cells were injected with N-terminal αB-crystallin antibody (S7 Fig). The fast migration phenotype of knocked down cells was rescued either by injection of purified αB-crystallin protein (S7 Fig) or by Rac1^V12^ expression (S8 Fig).

### αB-crystallin regulates cell polarity and adhesion stability through altered vinculin positioning

**Fig 11:**
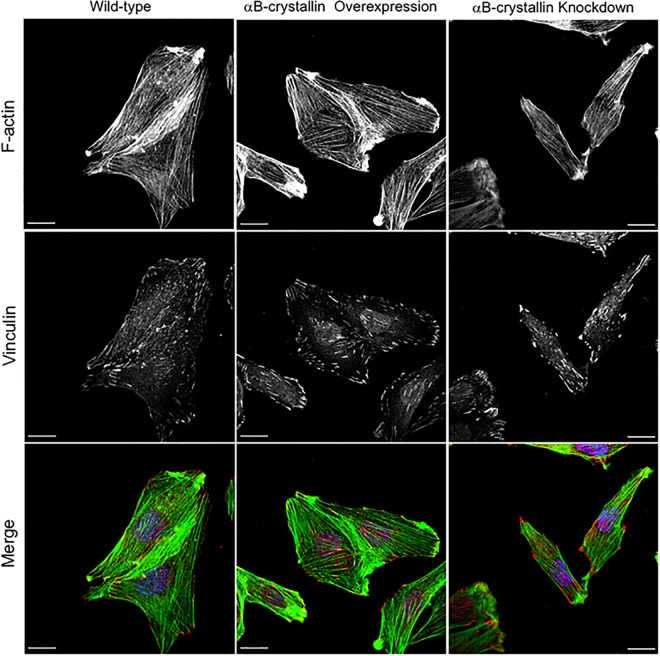
Focal adhesion-stress fiber phenotype is αB-crystallin dependent. Wild-type, αB-crystallin overexpressed, and knockdown L6 cells were visualized for F-actin (green), vinculin (red), and nucleus (blue). Bar is 20 μm.

**Fig 12:**
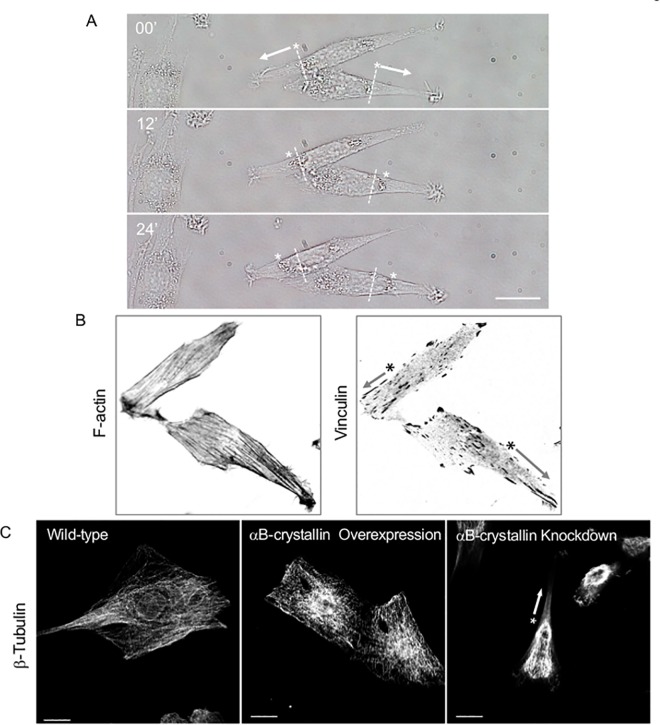
Time-lapse imaging of αB-crystallin knockdown L6 cells. Directional cell migration as fast as about 10 μm/ h are typically seen. Position of the nuclei are indicated by asterisks and arrows indicate the migratory direction. Bar is 20 μm (A). Asymmetric distribution of nucleus, F-actin stress fiber, and focal adhesion of αB-crystallin knockdown cells (B, inverted contrast image of Fig 11) are coincide with asymmetric polymerization of microtubule caused by *α*B-crystallin knockdown. Bar is 20 μm (C).

To clarify the mechanism where αB-crystallin is precisely involved for cell shaping and migration, vinculin as the essential component of actin stress fiber-focal adhesion was examined by immunofluorescence. Focal adhesion follows the maturation process from nascent integrin-actin interaction to myosin II-mediated focal adhesion maturation through paxillin phosphorylation of FAK [34]. During this process, vinculin which has over 14 binding partners, has to be activated from the auto-inhibitory self-binding state. Vinculin regulates directionality and cell polarity, and the knockdown showed reduced traction force and polarity loss [35]. As shown in Fig 11, well-developed actin stress fibers were confirmed independent of αB-crystallin expression but position of matured focal adhesion as visualized by vinculin immuno-staining, stress fiber direction, length, and density were clearly αB-crystallin dependent. To gain insight the structure-function correlation regarding αB-crystallin orchestrated focal adhesion, and the mode of knockdown cell migration was carefully examined by time-lapse live cell imaging. For successful cell migration, turnover of the focal adhesion is necessary and microtubule targeting may involve controlling disassembly [36]. As shown in Fig 12, position of nucleus was shifted nearly 10 μm within less than a half hour and consistent with the results as already described. Not leading edge but trailing end dramatically shrinked after the rapid disappearring of the active membrane ruffling where Rac-1 is involved, and the force pushed the nucleus place forward along with the cell body onward. We have been reported αB-crystallin as a molecular chaperon for tubulin/microtubule [11]. Knockdown of αB-crystallin dramatically altered the microtubule localization within the cell and showed a comet-like tail structure behind the nucleus where adhesion site turnover may occur (Fig 12C). Exact mechanism why microtubule is rather thick compare to wild-type are unknown but an assembled mixed microtubule structure formed when cellular equilibrium [αB-crystallin] << [tubulin] ratio may have stabilized the microtubule as previously proposed [29].

## Discussion

**Fig 13:**
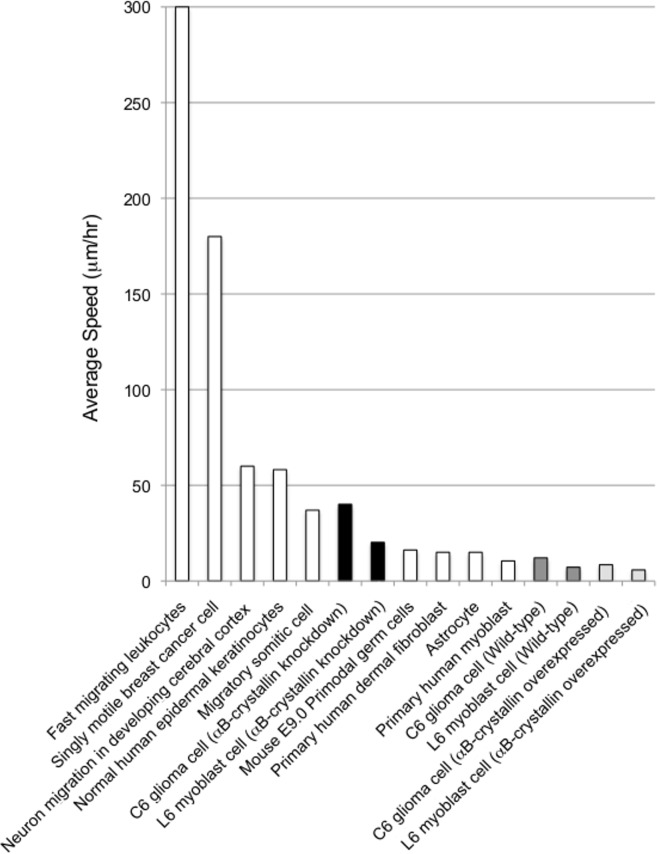
Average migration speed of cells. Fast migrating leukocytes (~300–1500 μm/hr.) [37]; single motile breast cancer cells (180 μm/hr) [38]; neuron migration in developing cerebral cortex (~60 μm/hr) [39]; normal human epidermal keratinocytes (58.2±2.4 μm/hr) [40]; migratory somatic cells (37±15 μm/hr) [41]; αB-crystallin knockdown C6 glioma cells (40.1 μm/hr, this study); αB-crystallin knockdown L6 myoblast cells (20.2 μm/hr, this study); mouse E9.0 primordial germ cells (16.2±2.5 μm/hr) [42]; primary human dermal fibroblasts (15 μm/hr) [43]; astrocytes (15 μm/hr) [44]; primary human myoblasts (10.5±5.8 μm/hr) [45]; C6 glioma cell (12.1 μm/hr, this study); L6 myoblast cells (7.2 μm/hr, this study); αB-crystallin-overexpressing C6 glioma cells (8.5 μm/hr, this study); αB-crystallin-overexpressing L6 myoblast cells (5.8 μm/hr, this study).

Migration speeds were compared to the reported values in the literature (Fig 13). From these results, the αB-crystallin-dependent cell shape alterations were found to dramatically affect the dynamics of cell adhesion. The αB-crystallin-dependent cell characteristics determined in this study are summarized in Tables 1 and 2 for C6 glioma cells and L6 myoblast cells, respectively.

**Table 1:** αB-crystallin-dependent cell characteristics in rat C6 glioma cells.

	Wild-type C6 cells	αB-crystallin overexpressing C6 cells	αB-crystallin knockdown C6 cells
Migration (μm/h)	12.1	8.5	40.1*
Area (μm^2^)	1.22×10^3^	2.93×10^3^*	0.99×10^3^*
Radius standard deviation (%)	32.6	28.1*	48.5*
Eccentricity	0.870	0.807	0.959*
Shape Index	0.323	0.362*	0.126*
Cell division(h)	24.3	52.2*	49.2*
Area of the layered cell at 80% confluency(%)	5.4	4.8	13.2*

*Significant, compare to wild type, n = 500.

**Table 2:** αB-crystallin dependent cell characteristics in rat L6 myoblast cells.

L6	Wild-type L6 cells	αB-crystallin overexpressed L6 cells	αB-crystallin knockdown L6 cells
Migration (μm/h)	7.2	5.8	20.2*
Area (μm^2^)	1.42×10^3^	1.32×10^3^	1.45×10^3^
Radius standard deviation (%)	25.8	20.5*	39.2*
Eccentricity	0.822	0.759*	0.895*
Shape Index	0.319	0.382*	0.199*
Cell division(h)	23.8	84.2*	37.4*
Area of the layered cell at 80% confluency(%)	2.9	3.2	11.5*

*Significant, compare to wild type, n = 300.

It has been well known for decades that cell shape controls cellular function [46, 47]. The cell is dynamic in nature and epigenetically adaptable to the environment through mechano-transduction [48] and cytoskeletal dynamics [49, 50]. In this study, we showed that a small HSP, αB-crystallin, is necessary for maintenance of its normal cell shape and persistent adhesion under physiological conditions (no added stress). Although small HSPs are known for their association with cytoskeletal proteins, this is the first report that provides direct evidence that chaperons impact cell shape and adhesion at physiological condition. Interaction between focal adhesion kinase (FAK) and αB-crystallin may be involved in this process because Pereira et al. recently reported that αB-crystallin directly bound to and protected FAK from calpain degradation in cardiomyocytes under stress conditions [12]. In this report, we showed that two different cell lines, a myogenic cell line, L6, as a muscle model [51] and C6, a differentiated rat brain tumor cell line [52], were susceptible to changes in the cytoplasmic levels of αB-crystallin.

Both overexpression and knockdown of cytoplasmic αB-crystallin led to cell shape alterations and therefore affected the maintenance of adhesion. The fact that the quantity of αB-crystallin clearly altered cell morphology on a culture dish in the absence of severe stress (heat or oxidative stress) indicates that the roles played by αB-crystallin are physiologically relevant for cell structure and function. Cell adhesion was dramatically enhanced by excess αB-crystallin and greatly reduced by lower levels of αB-crystallin. Knock down of αB-crystallin altered cellular migration. This was due to changes in substrate attachment, which may involve transient increases in [Ca^2+^]i by activation of stretch-responsive channels and/or mechano-sensitive channels on the membrane. It is also possible that disruption of the linkage between microtubule and microfilament systems was affected [53, 54, 55], leading to disassembly of the adhesion complex through calpain activation [12, 56].

The possible involvement of Rac1 in the microtubule-αB-crystallin adhesion complex and the membrane domain is highly plausible. Although αB-crystallin is a soluble protein, increasing evidence has shown that it can function as a membrane-associated protein. For example, it plays the following roles: (1) a Golgi-associated membrane protein in the lens [57], (2) in fiber cell membranes in the lens [58], (3) enhanced mitochondrial association upon oxidative stress [59], (4) ER anchoring [60], (5) stress-induced αB-crystallin-Ser59 in the plasma membrane of dendrites [61], (6) exosomes [62] and (7) protection against mechanical stress-induced calpain-dependent degradation of FAK in cardiomyocytes [12]. Note that Pereira et. al. have not mentioned changes in cell shape [12], the elongated morphology of αB-crystallin-depleted cardiomyocytes following siRNA treatment is consistent with our results.

Recently, plectin-deficiency has been examined for its role in contractile forces generated by cell adhesion and cell motility both in myoblasts and keratinocytes [63]. The adhesion strength and traction force were weaker for plectin^-/-^ myoblast cells than wild-type cells. Nonetheless, the shape of the plectin^-/-^ myoblast cells was very similar to αB-crystallin knockdown cells, and the migration speed was not altered. Thus, plectin is not essential for the dynamic behavior of the cells. We showed that a constitutively active form of Rac1 (Rac^V12^) rescued the cell area knockdown phenotype of αB-crystallin, perhaps through FAK localization to lamellipodia [64]. Tubulin is a soluble protein and important for cell division and cell morphology. It also associates with membranes under both normal physiological conditions and stress conditions. Furthermore, it associates with the nuclear membrane [65], affects membrane and organelle dynamics with microtubule-associated motor proteins [66], alters mitochondrial function [67] [68] and is involved in stretch activation of regulatory subunits of NOX2 on transverse tubule (T-T) membranes of the heart muscle [69]. In addition, it has long been known that Rac1 functions with microtubules [70] [71], that membrane-associated Rac1 is a regulatory component of the NOX2 complex [72] and αB-crystallin participates in stretch-induced muscle contraction [12]. Thus, there is much more to be learned about αB-crystallin [1] [73] and its role in microtubule dynamics, mechano-biology and membrane physiology. Finally, we also found that αB-crystallin regulates cell polarity and adhesion stability through altered vinculin positioning. Knockdown of αB-crystallin dramatically altered the microtubule localization within the cell and affected the focal adhesion turnover leads to unstable adhesion. This study showed a direct evidence of a small heat shock protein, αB-crystallin, is not only important for health and diseases but also showed the protein is a part of the large complex of focal adhesion and play a key role in cell migration.

## Supporting Information

S1 Fig Primary glial cell from rat E18 embryos. αB-crystallin (upper) and α-tubulin (bottom) immunofluorescence images of GFAP-positive astrocyte. Fluorescence images were obtained using TCS-SP5 (Leica) equipped with x63 oil immersion lens. Bar is 50 μm.
(TIFF)

S2 Fig Relative amounts of αB-crystallin protein. In wild-type (WT), overexpressing (OE) and knockdown (KD) C6 glioma cells and L6 myoblast cells were analyzed by Western blotting using αB-crystallin antibody. After the primary antibody (anti-αB C1 rabbit polyclonal antibody) treatment [11, 19], blots were incubated with HRP-conjugated secondary antibody (Jackson ImmunoResearch Labs, West Grove, PA) and signals were detected using the ECL system (Amersham Biosciences UK). The result of densitometry analysis shows these differences in the expression levels of αB-crystallin, where wild-type is 1.
(TIFF)

S3 Fig Morphological characteristics. Immunofluorescence images of αB-crystallin and actin antibody staining of L6 and C6 cells (upper). Bar is 20 μm. 3D downward view drawing of the phase-contrast observations of C6 wild type, αB-crystallin-overexpressing and knockdown cells (bottom). The αB-crystallin knockdown cell appeared to be thinner in two-dimensions but thicker if the volume of the object was determined by the relative refractive index (bottom).
(TIFF)

S4 Fig Phase-contrast images of αB-crystallin siRNA knockdown cells. Short interference double stranded RNA expression for knockdown αB-crystallin in L6 cells. MISSION siRNA Universal Negative Control (SIGMA-Aldrich) and double-stranded RNA targeting 5’rCUGUGAACCUGGACGUGA3’ of *Rattus norvegicus* αB-crystallin (NM_012935.3) were purchased from Sigma Genosys (Japan), introduced into the cell, and observed after 3 hr incubation at 37°C.
(TIFF)

S5 Fig Microinjection for evaluating αB-crystallin phenotypic revertants. See Material and Method section for details. Asterisk indicates the presence of fluorescence derived from Alexa 488 as an injection marker.
(TIFF)

S6 Fig Flow cytometric analysis of C6 cells. Results are summarized in graph (Fig 3B).
(TIFF)

S7 Fig Phenotypic assay. Wild-type, αB-crystallin-overexpressing and knockdown cells were injected with αB-crystallin antibody (top) and αB-crystallin protein (bottom), and cell migration speed (μm/ hr) was measured after three to five hours. * = P<0.05, n = 50.
(TIFF)

S8 Fig Phenotypic assay. Cell migration speed of EGFP-Rac1-, Rac1^V12^- and Rac1^N17^-expressing wild-type, αB-crystallin overexpressing and knockdown C6 cells. **, P<0.01; *, P<0.05; n = 50.
(TIFF)
