# Supplementary figures and images for: Small Heat Shock Protein αB-Crystallin Controls Shape and Adhesion of Glioma and Myoblast Cells in the Absence of Stress
Source: PLoS One. 2016 Dec 15;11(12):e0168136. doi: 10.1371/journal.pone.0168136 (PMC5158045; doi:10.1371/journal.pone.0168136)

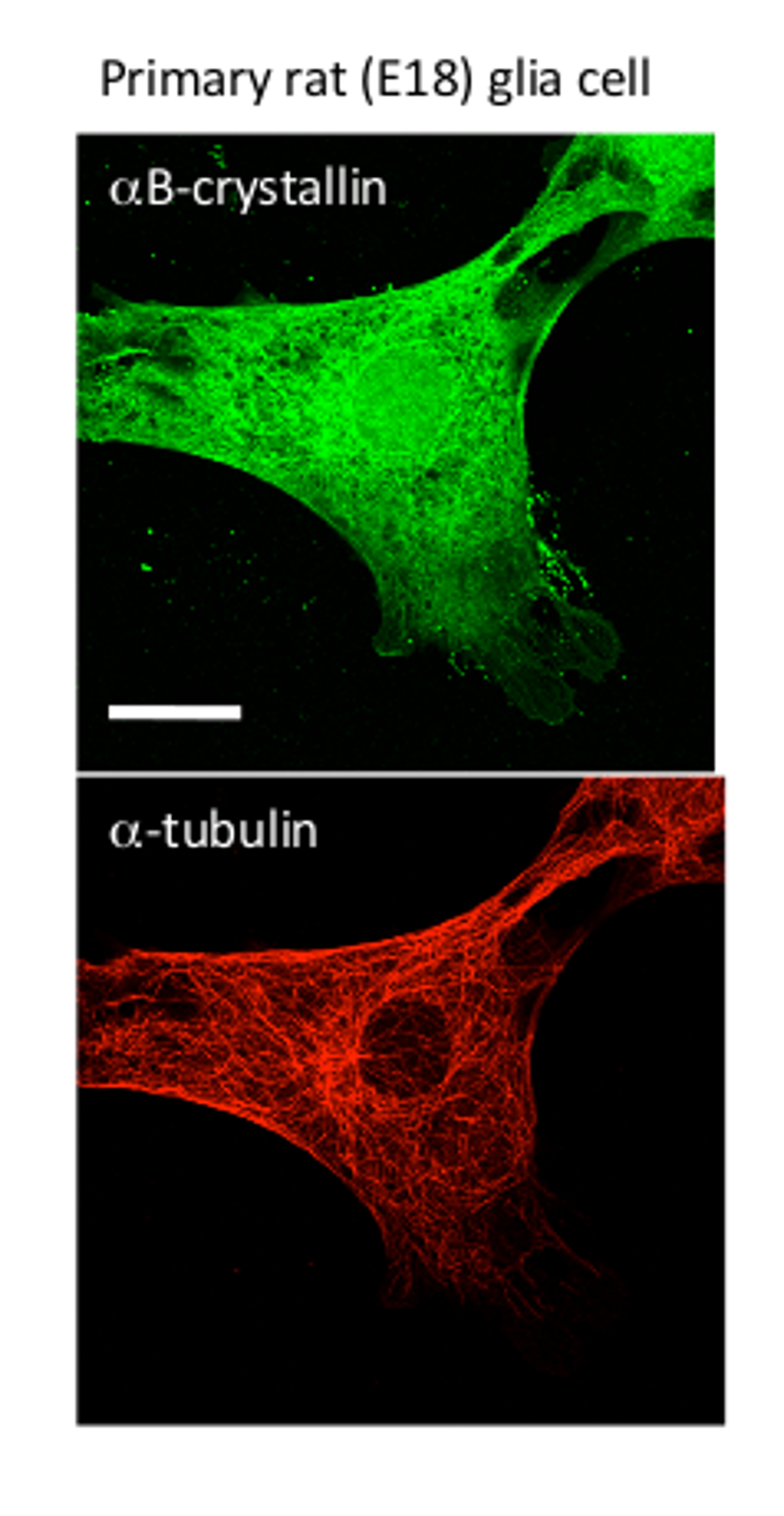

Supplement: S1 Fig — αB-crystallin (upper) and α-tubulin (bottom) immunofluorescence images of GFAP-positive astrocyte. Fluorescence images were obtained using TCS-SP5 (Leica) equipped with x63 oil immersion lens. Bar is 50 μm. (TIFF) [file pone.0168136.s001.tiff]

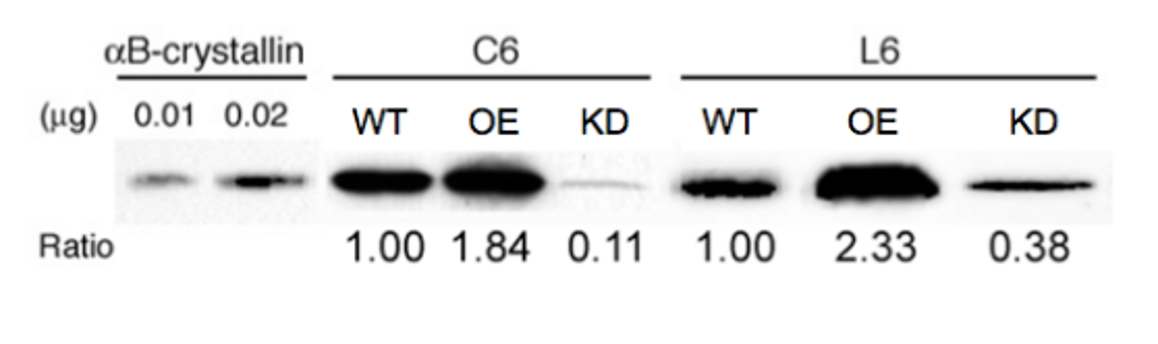

Supplement: S2 Fig — In wild-type (WT), overexpressing (OE) and knockdown (KD) C6 glioma cells and L6 myoblast cells were analyzed by Western blotting using αB-crystallin antibody. After the primary antibody (anti-αB C1 rabbit polyclonal antibody) treatment [11, 19], blots were incubated with HRP-conjugated secondary antibody (Jackson ImmunoResearch Labs, West Grove, PA) and signals were detected using the ECL system (Amersham Biosciences UK). The result of densitometry analysis shows these differences in the expression levels of αB-crystallin, where wild-type is 1. (TIFF) [file pone.0168136.s002.tiff]

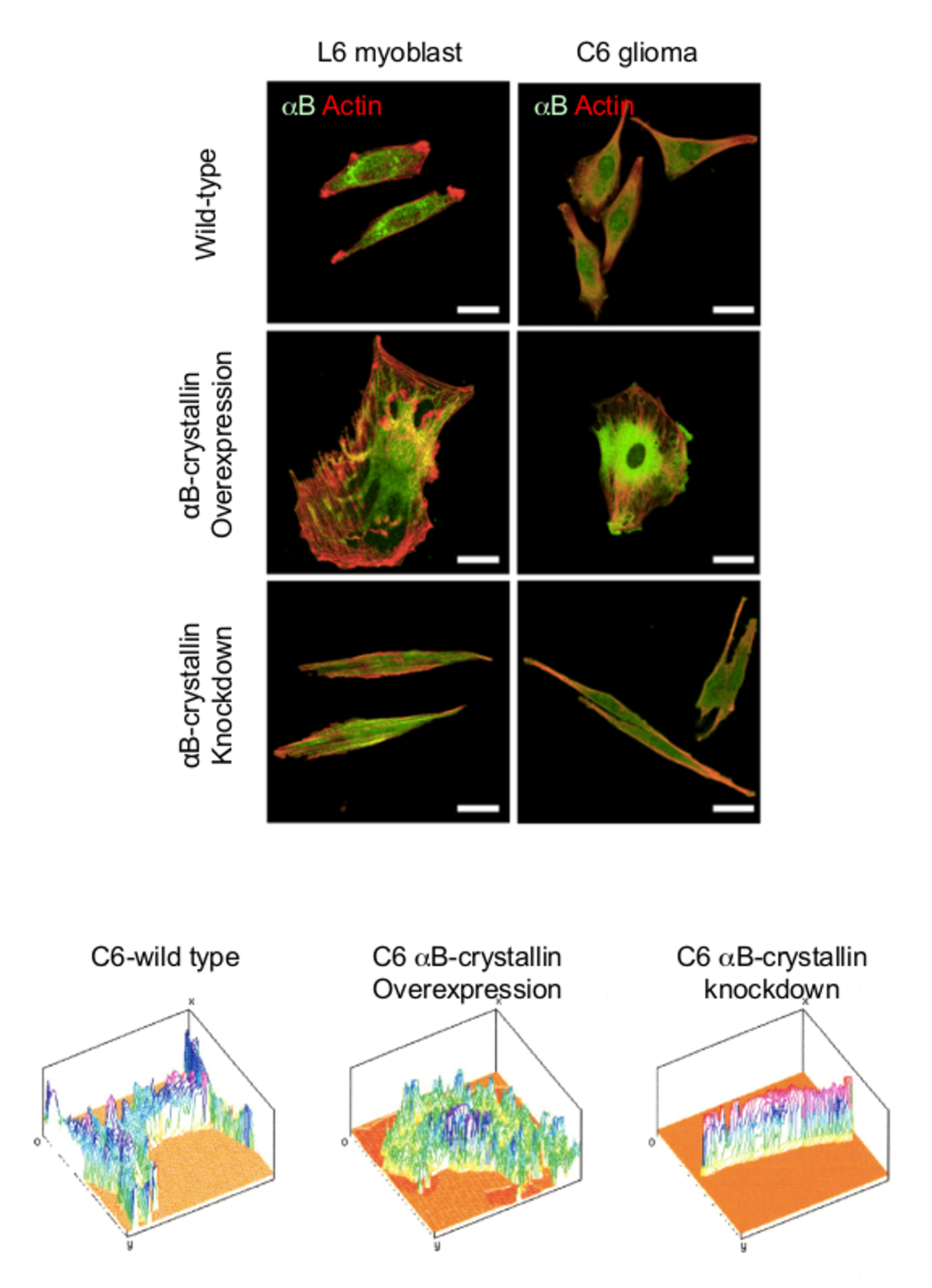

Supplement: S3 Fig — Immunofluorescence images of αB-crystallin and actin antibody staining of L6 and C6 cells (upper). Bar is 20 μm. 3D downward view drawing of the phase-contrast observations of C6 wild type, αB-crystallin-overexpressing and knockdown cells (bottom). The αB-crystallin knockdown cell appeared to be thinner in two-dimensions but thicker if the volume of the object was determined by the relative refractive index (bottom). (TIFF) [file pone.0168136.s003.tiff]

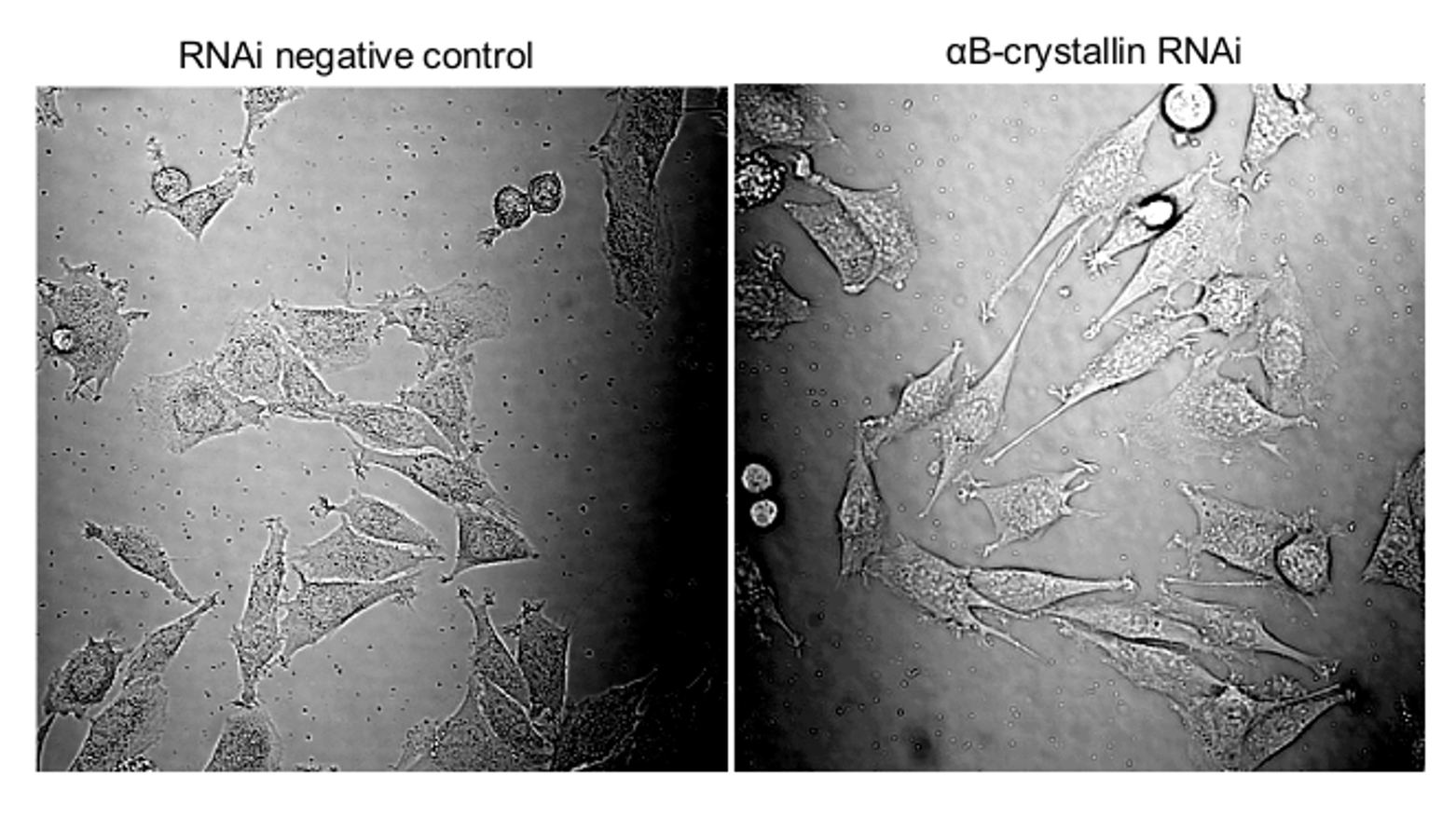

Supplement: S4 Fig — Short interference double stranded RNA expression for knockdown αB-crystallin in L6 cells. MISSION siRNA Universal Negative Control (SIGMA-Aldrich) and double-stranded RNA targeting 5’rCUGUGAACCUGGACGUGA3’ of Rattus norvegicus αB-crystallin (NM_012935.3) were purchased from Sigma Genosys (Japan), introduced into the cell, and observed after 3 hr incubation at 37°C. (TIFF) [file pone.0168136.s004.tiff]

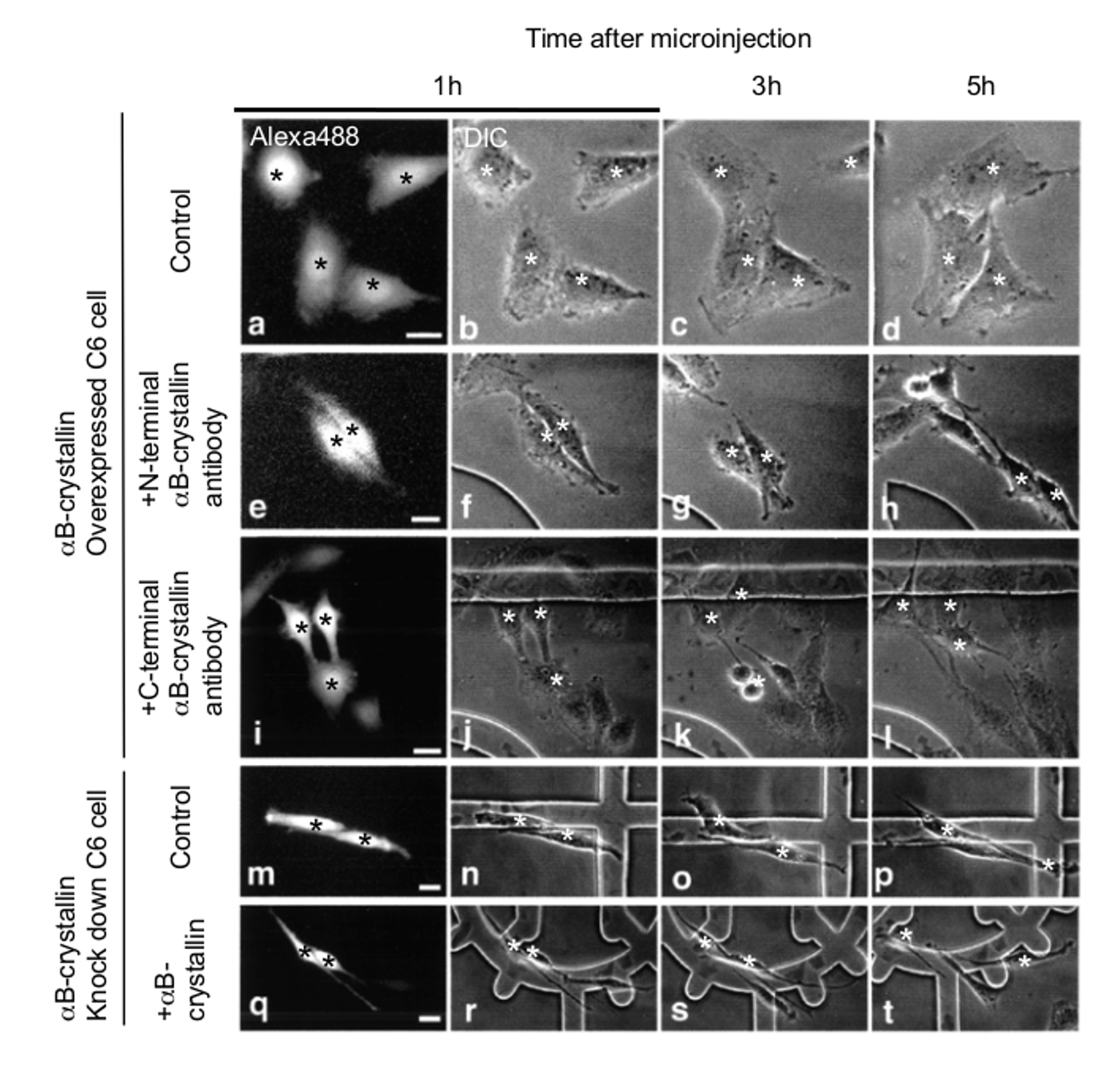

Supplement: S5 Fig — See Material and Method section for details. Asterisk indicates the presence of fluorescence derived from Alexa 488 as an injection marker. (TIFF) [file pone.0168136.s005.tiff]

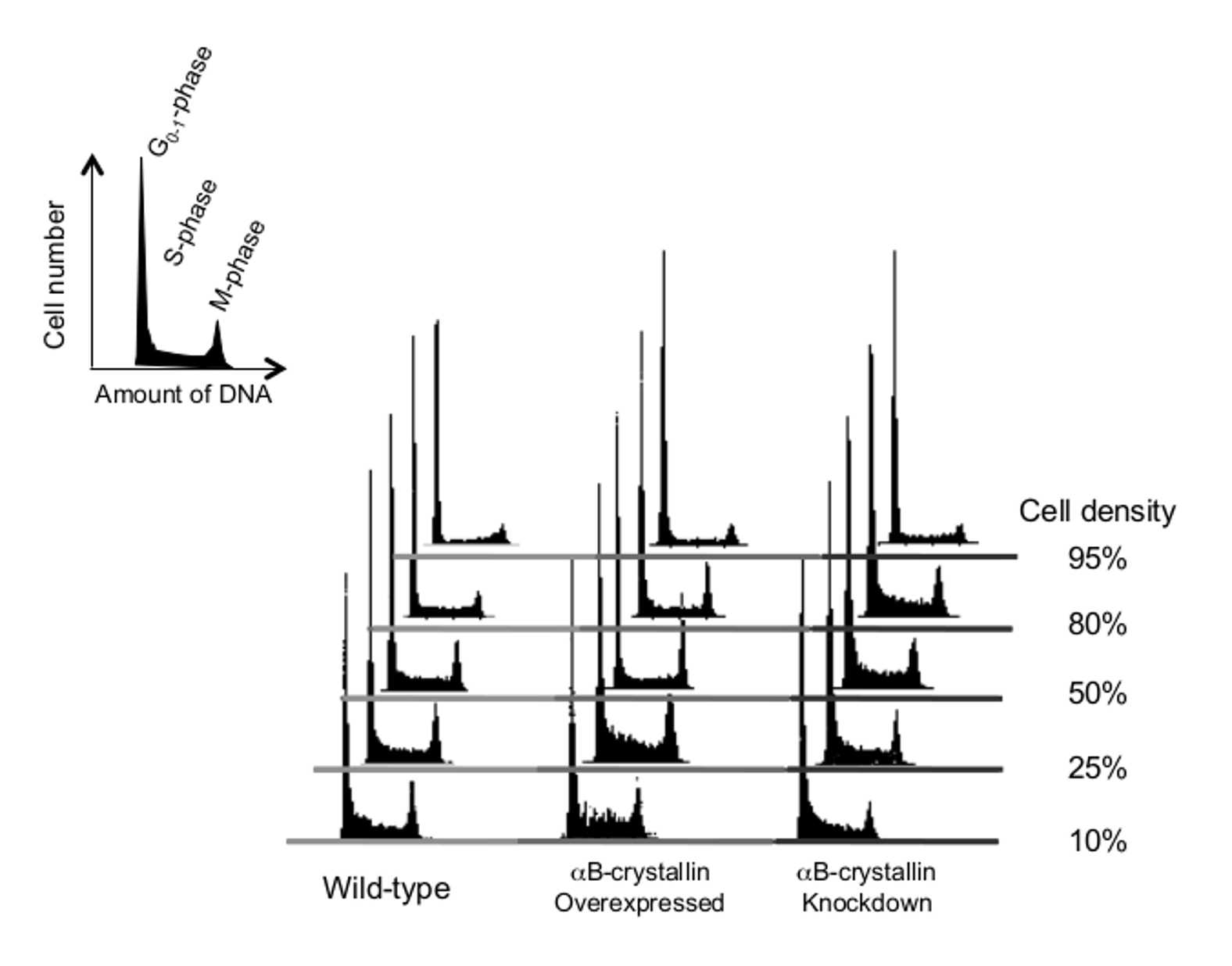

Supplement: S6 Fig — Results are summarized in graph (Fig 3B). (TIFF) [file pone.0168136.s006.tiff]

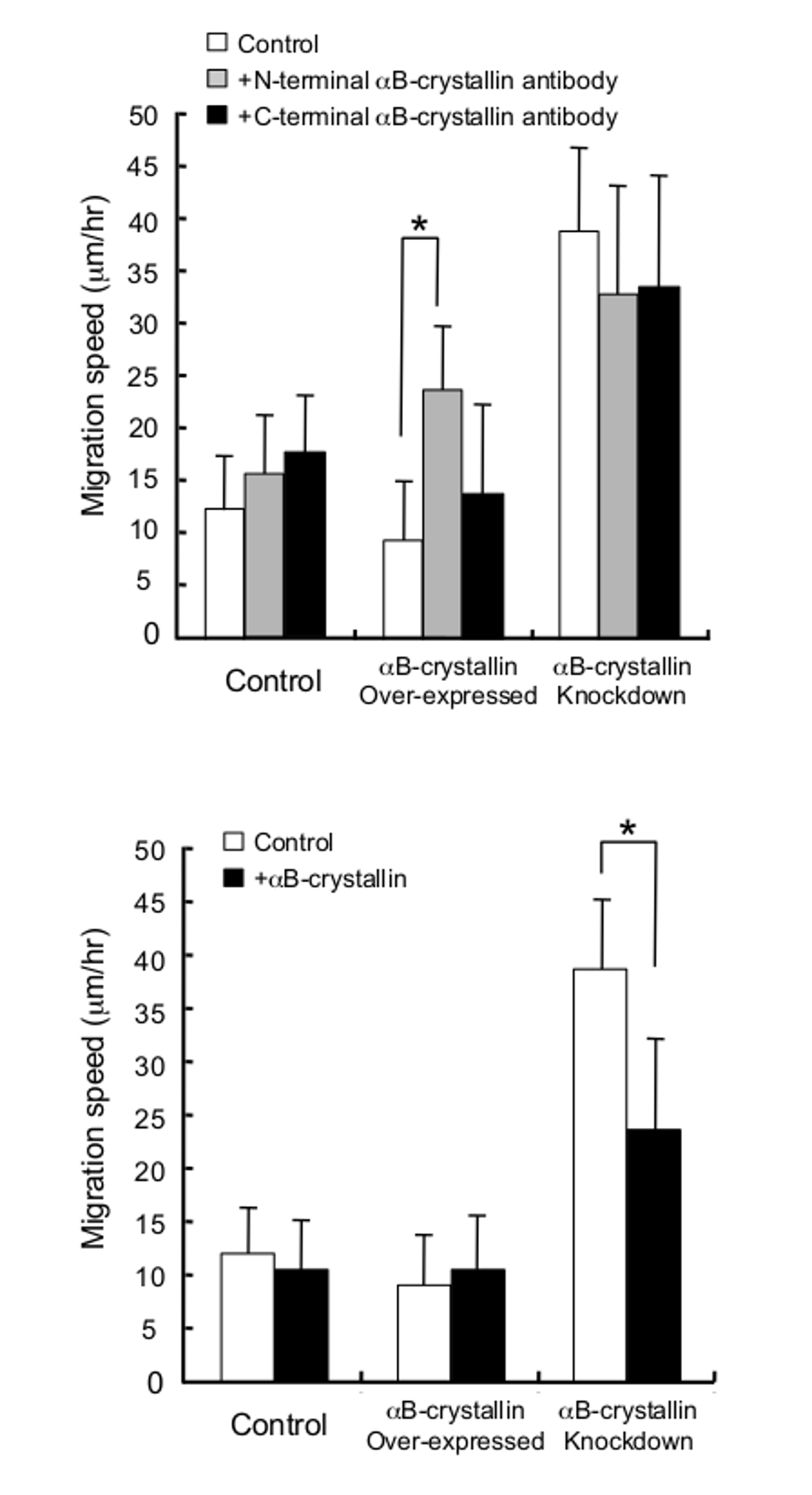

Supplement: S7 Fig — Wild-type, αB-crystallin-overexpressing and knockdown cells were injected with αB-crystallin antibody (top) and αB-crystallin protein (bottom), and cell migration speed (μm/ hr) was measured after three to five hours. * = P<0.05, n = 50. (TIFF) [file pone.0168136.s007.tiff]

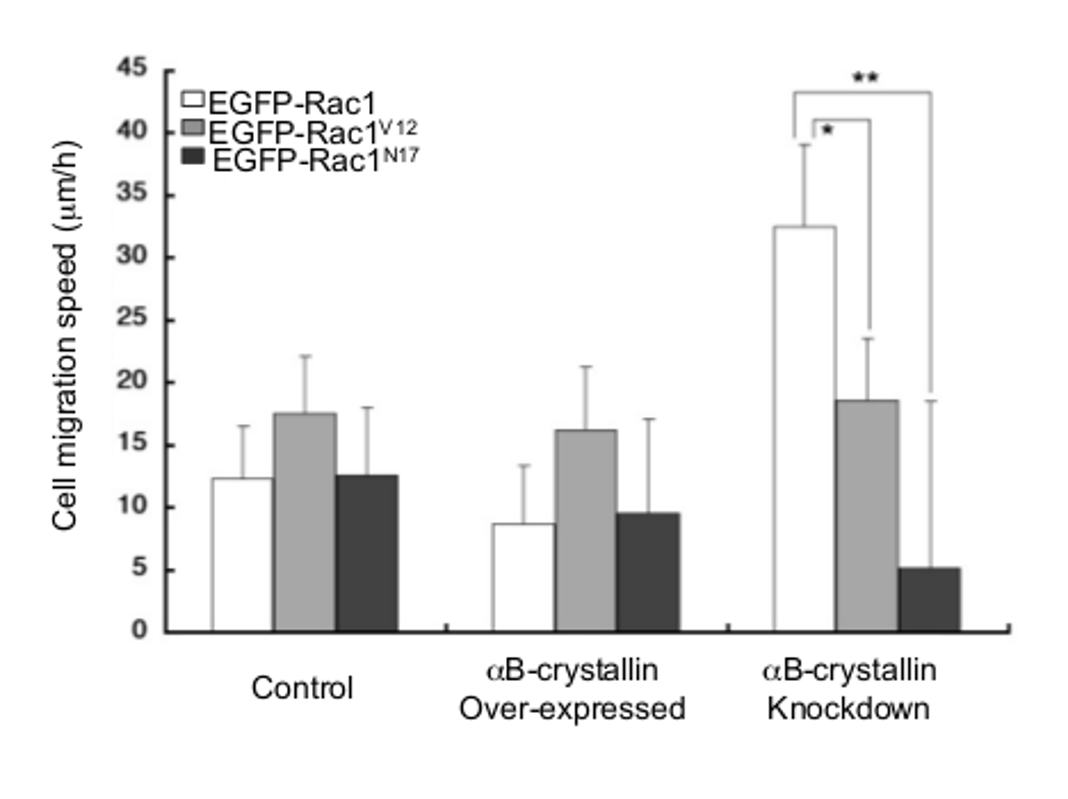

Supplement: S8 Fig — Cell migration speed of EGFP-Rac1-, Rac1V12- and Rac1N17-expressing wild-type, αB-crystallin overexpressing and knockdown C6 cells. **, P<0.01; *, P<0.05; n = 50. (TIFF) [file pone.0168136.s008.tiff]
